# Inhibition of extracellular proteases improves the production of a xylanase in *Parageobacillus thermoglucosidasius*

**DOI:** 10.1186/s12896-019-0511-0

**Published:** 2019-03-20

**Authors:** Alexandria T. N. Holland, Michael J. Danson, Albert Bolhuis

**Affiliations:** 1 Department of Pharmacy and Pharmacology, University of Bath, Claverton Down, Bath, BA2 7AY UK; 2 Department of Biology and Biochemistry, University of Bath, Claverton Down, Bath, BA2 7AY UK; 3 Present address: Centre for Biomolecular Sciences, University of Nottingham, Nottingham, UK

**Keywords:** *Parageobacillus thermogluosidasius*, Protein secretion, Xylanase, Protease inhibition

## Abstract

**Background:**

*Parageobacillus thermoglucosidasius* is a thermophilic and ethanol-producing bacterium capable of utilising both hexose and pentose sugars for fermentation. The organism has been proposed to be a suitable organism for the production of bioethanol from lignocellulosic feedstocks. These feedstocks may be difficult to degrade, and a potential strategy to optimise this process is to engineer strains that secrete hydrolases that liberate increased amounts of sugars from those feedstocks. However, very little is known about protein transport in *P. thermoglucosidasius* and the limitations of that process, and as a first step we investigated whether there were bottlenecks in the secretion of a model protein.

**Results:**

A secretory enzyme, xylanase (XynA1), was produced with and without its signal peptide. Cell cultures were fractionated into cytoplasm, membrane, cell wall, and extracellular milieu protein extracts, which were analysed using immunoblotting and enzyme activity assays. The main bottleneck identified was proteolytic degradation of XynA1 during or after its translocation. A combination of mass spectrometry and bioinformatics indicated the presence of several proteases that might be involved in this process.

**Conclusion:**

The creation of protease-deficient strains may be beneficial towards the development of *P. thermoglucosidasius* as a platform organism for industrial processes.

**Electronic supplementary material:**

The online version of this article (10.1186/s12896-019-0511-0) contains supplementary material, which is available to authorized users.

## Background

(Para)geobacilli and other thermophilic bacilli are of interest, firstly, as platform organisms for industrial processes. Thermophiles that grow optimally above 50 °C have several potential benefits, such as being non-pathogenic, a reduced risk of contamination during fermentations as compared to mesophilic microbes, reduced costs for cooling in large-scale fermenters, and improved solubility of feedstocks such as lignocellulosic biomass [1, 2]. Secondly, these organisms are an important potential source of thermostable enzymes for various industrial applications such as for detergents, paper bleaching, baking, brewing, animal feed, and biofuels [3]. Many of these enzymes are secreted hydrolases, but the process of protein transport in these thermophiles has not been characterised in any detail, in particular when compared with mesophilic model organisms such as *Bacillus subtilis* or *Escherichia coli*. A few studies have described using *Parageobacillus* (formerly named *Geobacillus* spp.) and *Geobacillus* spp. as a host for protein secretion, such as glycosyl-hydrolase secretion in *Parageobacillus thermoglucosidasius* [4] and heterologous cellulase production in *Geobacillus kaustophilus* [5], which when expressed in *Escherichia coli* were insoluble. However, to make (Para)geobacillus more attractive as a platform organism a better understanding of protein secretion is desirable. This could have a significant impact on, for instance, consolidated bioprocessing [6, 7], which is a process in which microbes are able to release sugars from cheap lignocellulosic feedstocks by producing and secreting hydrolytic enzymes that can degrade that feedstock. The sugars released can then, in turn, be converted into valuable products such as bioethanol or biobutanol [3].

In most bacteria, the majority of proteins are secreted in a post-translational fashion through the Sec system [8]. This facilitates the translocation of proteins through the SecYEG translocon, with the energy for this pathway provided by the ATPase SecA. All proteins utilising this pathway are synthesised as pre-proteins with a transient N-terminal signal peptide of around 16–30 amino acids that is removed during the transport process by signal peptidases. During or after secretion through the Sec system, proteins fold into their active conformation and either diffuse into the extracellular milieu or are retained at the cell envelope through specific interactions with the cytoplasmic membrane or cell wall [8]. In particular when proteins are overproduced, the translocation process may go wrong due to, for instance, limited capacity of the various components involved in the secretion process. For example, proteins could aggregate at the membrane or fold too slowly after release from the Sec translocon and become a target for hydrolysis by quality control proteases [9–11].

As a first step towards better understanding of the secretion process in *P. thermoglucosidasius*, we set out to investigate whether there are any bottlenecks in the secretion of a model protein. For this we chose XynA1 from *P. thermoglucosidasius*, a xylanase that is natively produced by *P. thermoglucosidasius* C56-YS93 [12]. An important reason to choose this enzyme is that it is easy to monitor using activity assays and immunoblotting. It was desirable to have plasmid-borne production only, as that would make it easier to monitor effects of e.g. changes in the primary sequence of XynA1. However, C56-YS93 is poorly transformable, and we used *P. thermoglucosidasius* TM242 [13] instead. The latter does not produce XynA1 and is genetically amenable, allowing us to monitor plasmid-produced xylanase.

## Results

**Fig. 1:**
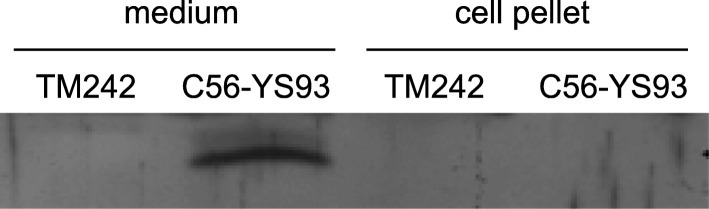
Western blot analysis of XynA1. The panel shows, on the left hand side, XynA1 secreted into the supernatant (medium), while the right hand side indicates cellular levels in strains TM242 and C56-YS93

Polyclonal antibodies recognising XynA1 were tested using P*. thermoglucosidasium* C56-YS93 and compared to *P. thermoglucosidasius* TM242, the latter of which does not contain the *xynA1* gene. As shown in Fig. 1, XynA1 is indeed only secreted into the medium by C56-YS93, while the protein is absent in TM242. The *xynA1* gene from *P. thermoglucosidasius* C56-YS93 was then cloned in a plasmid that replicates in *P. thermoglucosidasius* with and without the sequence encoding its signal peptide. This resulted in plasmids pXynA1 and pXynA1Δsp, respectively, and *P. thermoglucosidasius* TM242 was transformed with these plasmids. The TM242 strain was chosen for this as the C56-YS93 strain is poorly transformable with the plasmids we used in this study. The production of plasmid-borne XynA1 in TM242 was approximately two-fold greater than native xylanase production in C56-YS93 as estimated by Western blotting (data not shown).

**Fig. 2:**
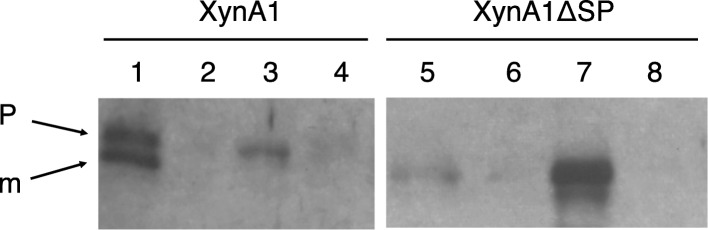
Western blot analysis of TM242 containing plasmid-borne XynA1. Left panel: TM242 containing plasmid pXynA1 (lanes 1–4); right panel: TM242 containing plasmid pXynA1Δsp (lanes 5–8). Culture medium: lanes 1 and 5; cell wall fraction: lanes 2 and 6; cytoplasmic fraction: lanes 3 and 7; membrane fraction: lanes 4 and 8. The positions of precursor (p) and mature (m) protein are indicated

Western blotting was used to visualise XynA1 with and without its signal peptide in cellular fractions. As shown in Fig. 2, XynA1 can be observed in *P. thermoglucosidasius* TM242 producing plasmid-borne xylanase, while this band was absent in TM242 without plasmid (Fig. 1). As expected, the mature form of XynA1 (indicated with the arrow “m”) is mainly present in the growth medium (Fig. 2, lane 1). Some precursor of XynA1 (pre-XynA1, indicated with the arrow “p”) is also in the growth medium, and this is most likely due to cellular lysis that we frequently observe with *P. thermoglucosidasius*. Precursor is also seen in the cytoplasmic fraction (lane 3), but the cell wall (lane 2) and membrane (lane 4) fractions contain very little XynA1.

In *P. thermoglucosidasius* TM242 containing plasmid pXynA1Δsp, the main bands run at the position of mature XynA1. A significant amount is found in the cytoplasmic fraction (lane 7), but some can also be observed in the medium fraction (lane 5). As mentioned above, the latter is probably due to the fact that *P. thermoglucosidasius* is prone to cell lysis. Nevertheless, results are as expected with the majority of XynA1 being secreted into the medium, and XynA1Δsp remaining in the cytoplasm, thus validating the fractionation procedure.

**Fig. 3:**
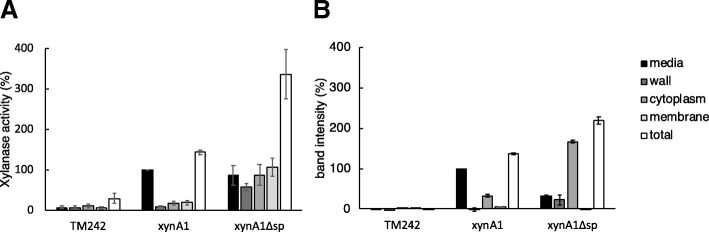
Quantification of the xylanase activity (**a**) and band intensity of Western blots (**b**) in media, cell wall, cytoplasmic and membrane fractions of *P. thermoglucosidasius* TM242 (without plasmid), and TM242 producing XynA1 or XynA1Δsp. Activity values are corrected for differences in OD_600_ between the cultures, although it should be noted that those differences were small. All enzyme activity and band intensity data are relative to the values in the media fraction of TM242 producing XynA1, which was set at 100% (black bars; XynA1). The error bars show the standard errors of the mean of 6 independent biological replicates

The amount of XynA1 protein was quantified in Western blots by measuring band intensity using ImageJ software, and this was compared to enzyme activity measured with the chromogenic substrate AZCL-xylan. Both measurements followed the same trend (Fig. 3), but some differences could also be observed. Firstly, the xylanase activity in the cytoplasmic fraction of the strain producing XynA1Δsp was low when compared to the levels observed by immunoblotting, indicating that some of the cytoplasmic material in the strain producing XynA1Δsp is not active. Secondly, for reasons that are not clear we did measure XynA1 activity in the membrane fraction, while the amount detected by Western blotting was very low.

**Fig. 4:**
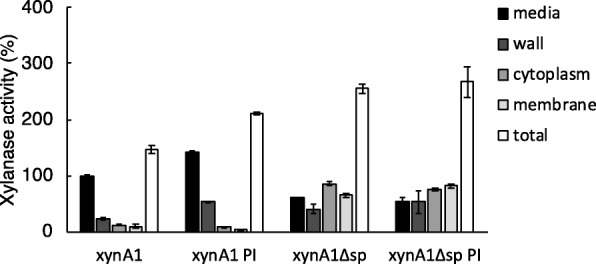
Xylanase activity in media, cell wall, cytoplasmic and membrane fractions of P*. thermoglucosidasius* TM242 producing XynA1 or XynA1Δsp, in the presence (PI) or absence of a protease inhibitor cocktail. Activity values are corrected for differences in OD_600_ between the *cultures* although it should be noted that those differences were small. All activity and band intensity data are relative to the values in the media fraction of TM242 producing XynA1, which was set at 100% (black bars; XynA1). The error bars show the standard errors of the mean of 3 independent biological replicates

Surprisingly, the xylanase activity in the medium fractions in the strains producing XynA1 and XynA1Δsp is very similar, with the latter only slightly (~ 15%) lower. However, the total amount of protein produced in the XynA1Δsp strain was 2.3-fold higher as compared to the strain producing XynA1; extracellular activity in the former represents ~ 25% of the total activity, whereas in the XynA1 strain this is ~ 70%. It seems thus very likely that the latter is due to genuine secretion, whereas the extracellular activity in the XynA1Δsp-producing strain is most likely due to the aforementioned problem of cellular lysis of *P. thermoglucosidasius*. The question remains why the total activity as well as the amount of protein as quantified by Western blotting was higher in the strain producing XynA1Δsp as compared to the strain producing XynA1 (2.3 and 1.6-fold, respectively). A two-sample *t*-test showed these differences to be significant (*p* < 0.05). We hypothesised that this was due to extracytoplasmic proteases that degrade XynA1 during or after its translocation. To test this, *P. thermoglucosidasius* TM242 (pXynA1) was grown in the presence of a cocktail of protease inhibitors. The cocktail used (cOmplete™ – EDTA free) inhibits a range of serine and cysteine proteases; this cocktail was chosen as it is not inhibitory to growth of *P. thermoglucosidasius*. In contrast, a cocktail with EDTA, which also inhibits metalloproteases, did affect the rate of growth and was therefore not suitable for our studies. As shown in Fig. 4, the addition of the protease inhibitors increased the XynA1 activity 1.4-fold (*t*-test: *p* < 0.05), and this increase was observed both in the medium and cell wall fraction. This clearly indicates that XynA1 is degraded by serine or cysteine proteases in the cell wall or extracellular milieu. In the strain producing XynA1Δsp, no significant differences in the xylanase activity were found, indicating that the difference in xylanase activity in the strains containing XynA1 and XynA1Δsp is the result of extracellular proteolysis.

The protease inhibitor experiment clearly shows that XynA1 is sensitive to degradation, and it would thus be of interest to identify extracellular proteases in *P. thermoglucosidasius*. To this purpose, we analysed the secreted proteome (secretome) of *P. thermoglucosidasius* C56-YS93 with a semi-quantitative shotgun mass spectrometry approach. It should be noted that, at the time of this analysis, the genome sequence of TM242 was not available, hence the proteome analysis of C56-YS93. When the genome sequence of TM242 became available, our bioinformatics analysis showed that the secretomes of TM242 and C56-YS93 differ very little (not shown), with only a few exceptions such as the absence of XynA1 in TM242. Identification of the proteins indicated that the samples were enriched in putative secretory proteins, but a significant proportion appeared to originate from the cytoplasm (data not shown). As indicated before, this is again due to the fact that *P. thermoglucosidadius* is prone to cell lysis when grown in laboratory conditions. Therefore, we combined this with a bioinformatics approach. We identified 82 putatively secretory proteins encoded by the genome of *P. thermoglucosidasius* C56-YS93 (Additional file 1 supplementary data, sheet 1). Of those signal peptide-containing proteins, 23 were also identified by mass spectrometry (Additional file 1, sheet 2). This data set includes seven putative proteases that are predicted to be secreted, and this was supplemented with three putative proteases that are membrane-bound (Table 1). Of the latter three, the lipoprotein AEH47729 was also identified by mass spectrometry. The secretory proteases are listed in order of abundance that their peptides were identified in the mass spectrometry approach taken, indicating that a subtilisin (a serine protease homologous with the AprE enzyme from *B. subtilis*) is the most abundant protease that is secreted by *P. thermoglucosidasius.* However, as the mass spectrometry method was, at best, semi-quantitative, we cannot make any statements on absolute quantities of subtilisin or any of the other proteases, or determine which protease is responsible for the degradation of XynA1. What we did note was that the majority of proteins identified are serine proteases, with only one being predicted to be a metalloprotease (GenBank ID AEH46439), corroborating our finding that the production of XynA1 can be improved using a protease inhibitor cocktail without EDTA.

**Table 1:** Putative proteases in *P. thermoglucosidasius* C56-YS93 that are secreted or located at thecell wall

Description	Predicted location	Genbank ID
Subtilisin	secreted	AEH46337
Extracellular zinc metallopeptidase, M23 family	secreted	AEH46439
Cell wall hydrolase/autolysin (Precursor)	secreted	AEH46389
Carboxyl-terminal protease	secreted	AEH46442
Serine-type D-Ala-D-Ala carboxypeptidase	secreted	AEH46091
Cell-wall bound hydrolase, containing NLP/P60 domain	secreted	AEH46339
Cell wall-associated hydrolase, NLP/P60 family	secreted	AEH47877
D-alanyl-D-alanine carboxypeptidase, vanY family	membrane (lipoprotein)	AEH47729
Peptidase S1 and S6 chymotrypsin/Hap^a^	transmembrane	AEH46370
HtrA2 peptidase^a^	transmembrane	AEH49728

^a^Not identified in the mass spectrometry analysis

An alternative strategy to overcome degradation would be to overproduce chaperones that enable the rapid folding of extracellular proteins such as XynA1. One obvious candidate for such a function is the extracytoplasmic lipoprotein PrsA. This protein is a prolyl isomerase, an enzyme that catalyses the *cis-trans* isomerisation of peptidyl-proline bonds in proteins. This is important for efficient and correct folding of proteins, and in *B. subtilis* cells in which PrsA is depleted there is increased degradation of secretory proteins [14]. Furthermore, overproduction of PrsA can enhance protein secretion for both native and heterologous proteins in *B. subtilis* [14, 15]. *P. thermoglucosidasius* TM242 also contains a gene encoding PrsA (GenBank ID ANZ29799). We tested whether co-expression of *prsA* with *xynA*1 improved the production of XynA1, but this did not have an effect (data not shown).

## Discussion

In this study we analysed the secretion of the enzyme XynA1 in the thermophile *P. thermoglucosidasius*. To monitor the secretion of this model enzyme we used Western blot analysis to determine the total amount of XynA1 produced, as well as activity assays. Both techniques largely showed the same trends when analysing different cellular fractions, indicating that most of the protein observed is also in its active conformation. However, some differences were observed for the strain producing XynA1 without its signal peptide. There was for instance, a lower level of xylanase activity in the cytoplasm then what would be expected from the amount of protein observed by Western blotting, indicating that some of the protein lacking its signal peptide is in an inactive conformation in the cytoplasm. This is not particularly surprising because Sec substrates such as XynA1 normally fold into their active conformation after translocation, a process that may be assisted by specific extracytoplasmic chaperones [16]. Those chaperones are absent in the cytoplasm, and it is therefore conceivable that some of the XynA1Δsp misfolds in the cytoplasm. Conversely, we observed that XynA1Δsp activity in the membrane fraction was considerably higher than could be expected from Western blotting. The reason for this is not clear, but it is possible that the detergent used to solubilise the membrane fraction (Triton X-100) enhances the activity of XynA1, either directly or by increasing accessibility of the substrate to enzymatic degradation. Stimulating effects of detergents on the activity of enzymes degrading lignocellulosic substrates have been observed before [17, 18], and we will investigate this for XynA1 in more detail in a future study.

Cellular lysis was observed in several of our experiments. For instance, 25% of the total amount of XynA1Δsp was found in the medium fraction, and several cytoplasmic proteins were identified in the mass spectrometry analysis. We speculate that this could be an artefact from growing *P. thermoglucosidasius* in laboratory conditions in which growth rates are relatively high, similar as observed for the related thermophile *Geobacillus thermoleovorans* [19].

An important observation was the significant increase in total xylanase activity and quantity when XynA1 was produced without its signal peptide. One reason for this could be due to differences in gene expression or mRNA stability of the strains used, even though their plasmids differ only in the sequence encoding the signal peptide. However, we hypothesised that the main reason for this was proteolytic degradation of XynA1 during or after its secretion. This was confirmed by a significant increase (1.4-fold) of xylanase activity in both medium and cell wall fraction when protease inhibitors were added to the culture medium, while these inhibitors had no impact on activity in the strain producing XynA1Δsp. These results were further corroborated by the presence of several extracytoplasmic proteases as identified by mass spectrometry and bioinformatics analysis. Based on the inhibitor cocktail used and proteases identified, at least one or more serine proteases in *P. thermoglucosidasius* appear to be involved in the degradation of XynA1. We did not test the importance of metalloproteases, as the metalloprotease inhibitor EDTA also inhibits growth of *P. thermoglucosidasius*.

In *B. subtilis,* the phenomenon of proteolytic degradation of secretory proteins is well described [20–23]. Similar to *P. thermoglucosidasius*, *B. subtilis* produces several secretory proteases, leading to high levels of proteolytic activity in the cell wall and extracellular milieu, making heterologous proteins in particular vulnerable to degradation. The slow folding of these proteins at the cell membrane-cell wall interface leaves them susceptible to hydrolysis by cell-wall associated proteases, as slowly-folding proteins expose protease-sensitive sites that are not exposed in the correctly-folded protein [14, 15, 24]. Misfolded or slowly-folding proteins are rapidly degraded to prevent interference with cell wall growth and renovation, and to prevent blockages at the translocase [25, 26]. Overproduction of extracyoplasmic chaperones such as PrsA may help in some cases [14, 15], but XynA1 activity was not improved upon co-expression of the *P. thermoglucosidasius prsA* gene. This indicates that PrsA levels are not limiting for XynA1 secretion in the conditions that we tested, or that XynA1 is not a substrate for PrsA in *P. thermoglucosidasius*.

At the secretion levels we obtained, no other significant bottlenecks were identified, although our study was to some extent limited by the lack of strong promoters that lead to the very high levels of protein secretion that can be achieved with *B. subtilis*. Therefore, the main way in which secretion of XynA1 can be improved is through inhibition of proteases. Here we showed that this can be achieved by addition of protease inhibitors, but that would be very costly if *P. thermoglucosidasius* would be used at an industrial scale. We are currently planning to generate protease-deficient strains of *P. thermoglucosidasius*, a strategy that has been shown to work for the production of heterologous proteins in *B. subtilis* [27–30]. Creating such strains would answer the question as to which protease is responsible for the degradation of XynA1. Furthermore, we plan to test whether production of XynA1 is sufficient for growth on a medium with xylan as the sole carbon source, and whether such growth can be improved by the use of protease knock-out strains.

## Conclusion

In summary, we show here that the main bottleneck in the secretion of XynA1 by *P. thermoglucosidasius* is proteolytic degradation during or after its translocation through the membrane. Inhibition of extracellular protease activity did increase the amount of secreted XynA1, and this provides a strategy to create strains that make more effective use of lignocellulosic feedstocks. More generally, protease-deficient strains are likely to be useful for the further development of *P. thermoglucosidasius* as a platform organism for industrial processes.

## Methods

### Bacterial strains and growth conditions

The strains used in this study are *P. thermoglucosidasius* TM242 [13] and *P. thermoglucosidasius* C56-YS93 [12]. Strains were routinely grown on TGP agar [13], whereas for all other experiments they were cultured in ammonium salts medium (ASM) [4] supplemented with 0.5% (*w*/*v*) yeast extract (Oxoid) and 0.5% (w/v) glucose. All chemicals were from Sigma Aldrich unless noted otherwise.

### Cloning of the xylanase-encoding gene

The xylanase gene (*xynA1*; GenBank ID AEH48179) was amplified from *P. thermoglucosidasius* C56-YS93 chromosomal DNA by PCR using oligonucleotides Xylbstbfor (aaaaattcgaaTGCGGAACGTTTTACGC; nucleotides identical to the genomic template DNA are in upper case, and restriction sites used for cloning are underlined) and Xylsac1rev (GCGGACGAGCTCTTATTTATGATCGATAATGGC). The *xynA1* gene without the region encoding its signal peptide was amplified using oligonucleotides GHspF (ttcgaaATGGCAGATACGGCTTCCTAT) and Xylsac1rev. PCR products were then digested with *Bst*BI and *Sac*I and ligated into the *Cla*I and *Sac*I restriction sites of the shuttle vector pUCG4.8-RPLS-sfGFP [31], thereby replacing the gene encoding green fluorescent protein with that of *xynA1*. The resulting plasmids were named pXynΑ1 and pXynΑ1Δsp, producing XynA1 with and without signal peptide, respectively. In both plasmids, the expression of the xylanase gene is under control of the sequence that controls transcription of the ribosomal protein RplS, which is a strong constitutive promoter [32]. The ligation mixtures were used to transform chemically competent *E. coli* JM109 cells. Colonies containing the correct inserts were selected, and plasmids were verified by sequencing. *P. thermoglucosidasius* TM242 was transformed with these plasmids using a previously described method [1].

### Antibodies

XynA1 with an hexahistidine tag, and without its signal peptide, was produced in *E. coli* and purified using affinity chromatography as described before [33]. Polyclonal antibodies were then raised in rabbits at the facilities of Eurogentec.

### Cell fractionation

*P. thermoglucosidasius* cells were grown on TGP agar to produce a thick lawn, which was then scraped off and added to 20 ml pre-warmed ASM. The cells were incubated at 60 °C with shaking at 220 rpm for 1 h, and a sample of the cell suspension was then diluted to an optical density at 600 nm (OD_600_) of 0.1 in 20 ml fresh pre-warmed ASM in 250 ml baffled conical flasks. The culture was then grown to an OD_600_ of around 1.5, and cells in 2 ml of the culture were harvested by centrifugation at 2000×*g*, with the supernatant stored as the medium fraction. The cell pellet was re-suspended in 2 ml pre-warmed protoplast buffer (20% sucrose, 50 mM Tris-HCl pH 7.5, 15 mM MgCl_2_, 5 μg/ml lysozyme) at 37 °C for 30 min. Protoplasts were then centrifuged at 700×*g* for 10 min. The supernatant was collected as the cell wall fraction, and the pellet as the protoplasts. The protoplasts were lysed by re-suspension in 2 ml 50 mM Tris-HCl pH 7.5 and sonication at 60 watts for 1 min on ice (Branson sonifier 250). After centrifugation at 50,000×*g* for 1 h (Beckman Coulter benchtop ultracentrifuge) the supernatant collected as the cytoplasmic fraction and the pellet (membrane fraction) was re-suspended in 2 ml 50 mM Tris-HCl pH 7.5 containing 0.1% Triton X-100.

### Western blot analysis of cell fractionation samples

Samples were resolved by SDS-PAGE with amounts loaded normalised based on the corresponding OD_600_ of the cell cultures. Proteins were transferred to polyvinylidene difluoride membrane (PVDF; Immobilon-P; Millipore) using a semi-dry transfer method as described [34]. Proteins were visualised with specific antibodies and the ECL western blotting substrate (Pierce), following the manufacturer’s instructions. Band intensity in Western blots was determined using ImageJ software.

### Xylanase activity assay

Unless otherwise stated, 8 mg/ml AZCL-xylan (Sigma) in phosphate citrate buffer at pH 7 was incubated with equal volumes (0.5 ml) of cell fractions for 1 h at 60 °C in 2 ml microfuge tubes. During incubation, samples were mixed on an end-over-end rotator. The tubes were then briefly centrifuged to remove insoluble xylan, and the absorbance of the supernatant was measured at 595 nm (BMG Labtech plate reader).

### Mass spectrometry

Proteins in the medium fraction of *P. thermoglucosidasius* C56-YS93 were resolved by SDS-PAGE, allowing the proteins to migrate only a short distance in the gel. The gel was then stained with Coommassie Brilliant Blue, and the gel chunk of interest was excised and subjected to in-gel digestion, using a ProGest Investigator in-gel digestion robot (Digilab) and following standard protocols [35]. The peptides were extracted with 10% (*v*/v) formic acid and separated on an Acclaim PepMap 100 C18 trap and an Acclaim PepMap RSLC C18 column (ThermoFisher Scientific), using a nanoLC Ultra 2D plus loading pump and nanoLC AS-2 auto sampler (Eskigent). The peptides were eluted with a gradient of increasing acetonitrile containing 0.1% (v/v) formic acid (5–40% acetonitrile in 5 min, 40–95% in a further 1 min, followed by 95% acetonitrile to clean the column, before re-equilibration to 5% acetonitrile). The eluent was sprayed into a TripleTOF 5600 electrospray tandem mass spectrometer (ABSciex) and analysed in Information Dependent Acquisition mode, performing 250 msec of MS followed by 100 msec MS/MS analyses on the 20 most intense peaks seen by MS. The MS/MS data file generated was analysed using the Mascot algorithm (Matrix Science) against the NCBI nr database (Aug 2013) with no species restriction, trypsin as the cleavage enzyme, carbamidomethyl as a fixed modification of cysteines, and methionine oxidation and deamidation of glutamines and asparagines as variable modifications.

### Bioinformatics

The open reading frames (ORFs) for *P. thermoglucosidasius* C56-YS93 was downloaded from NCBI and analysed using the SignalP 4.1 server [36] to identify ORFs that contain putative signal peptides. The ORFs that were positive for signal peptides were then analysed using the TMHMM [37] and LipoP [38] servers, which predict transmembrane domains and lipoprotein signal peptides, respectively. Proteins containing two or more transmembrane domains, or containing a lipopeptide signal peptide, were excluded from the list of secreted proteins.

## Additional file

Additional file 1: Secretory proteins encoded by the genome of *P. thermoglucosidasius* C56-YS93. Sheet 1: List of secretory proteins, identified by bioinformatics. Sheet 2: List of secretory proteins identified using both bioinformatics and mass spectrometry of the extracellular proteome. (XLSX 16 kb)

## Abbreviations

ASM: Ammonium salts medium
EDTA: Ethylenediaminetetraacetic acid
OD: Optical density
ORF: Open reading frame
PVDF: Polyvinylidene difluoride
